# Prognostic Significance of Immunohistochemical Markers and Genetic Alterations in Malignant Peripheral Nerve Sheath Tumors: A Systematic Review

**DOI:** 10.3389/fonc.2020.594069

**Published:** 2020-12-22

**Authors:** Enrico Martin, Ibtissam Acem, Dirk J. Grünhagen, Judith V. M. G. Bovée, Cornelis Verhoef

**Affiliations:** ^1^Department of Surgical Oncology, Erasmus Medical Center, Rotterdam, Netherlands; ^2^Department of Plastic and Reconstructive Surgery, University Medical Center Utrecht, Utrecht, Netherlands; ^3^Department of Pathology, Leiden University Medical Center, Leiden, Netherlands

**Keywords:** malignant peripheral nerve sheath tumors, prognosis, molecular, clinicopathologic, markers, genes

## Abstract

**Background:**

Malignant peripheral nerve sheath tumors (MPNSTs) are aggressive soft tissue sarcomas with dismal prognosis. Pathological and genetic markers may predict more aggressive behavior in MPNSTs but have uncommonly been investigated, and few are used in daily practice. This study reviews the prognostic value of immunohistochemical markers and genetic alterations in MPNST.

**Methods:**

A systematic search was performed in PubMed and Embase databases according to the PRISMA guidelines. Search terms related to ‘MPNST’ and ‘prognostic’ were used. Studies investigating the association of immunohistochemical markers or genetic alterations with prognosis were included. Qualitative synthesis was performed on all studies. A distinction was made between univariable and multivariable associations.

**Results:**

Forty-six studies were included after full-text screening. Sixty-seven different immunohistochemical markers were investigated. Absence of S100 and H3K27me3 and high Ki67 and p53 staining was most commonly independently associated with worse survival and disease-free survival. Several genetic alterations were investigated as well with varying association to survival. *TP53*, *CDK4, RASSF1A* alterations were independently associated with worse survival, as well as changes in chromosomal length in Xp, 10q, and 16p.

**Conclusions:**

MPNSTs harbor complex and heterogeneous biology. Immunohistochemical markers and genetic alterations have variable prognostic value. Absence of S100 and H3K27me3 and increased Ki67 can be of prognostic value. Alterations in *TP53* or increase in p53 staining may distinguish MPNSTs with worse outcomes. Genetic alterations and staining of other cell cycle regulatory and Ras pathway proteins may also help stratifying patients with worse outcomes. A combination of markers can increase the prognostic value.

## Introduction

Malignant peripheral nerve sheath tumors (MPNSTs) are rare and aggressive soft tissue sarcomas (STS) that carry a dismal prognosis (1–3). Neurofibromatosis type 1 (NF1) patients have an increased risk of developing these tumors and encompass approximately 25–50% of MPNST patients (1–5). The *NF1* gene is commonly affected in MPNSTs which causes loss of the neurofibromin protein which inhibits the Ras enzyme (6). Activation of the Ras pathway leads to upregulation of the mitogen-activated protein (MAPK) and phosphoinositide 3-kinase (PI3K) pathways (7). Besides the common knockdown of *NF1*, alterations in several genes including *TP53, SUZ12, EED, PTEN*, and *CDKN2A* as well as upregulation of several tyrosine kinases contribute to the formation of MPNST (8–12). MPNSTs are known for harboring complex genomic alterations, but despite our increasing understanding of underlying biology, prognosis has not ameliorated the past decades and median survival stagnates at 5–6 years (2, 3).

Staging of MPNSTs is important to increase accuracy of outcome prediction, but it may also facilitate treatment stratification. However, the clinical American Joint Committee of Cancer (AJCC) STS staging system is less applicable in MPNST (4, 5, 13). The histologic Fédération Nationale des Centres de Lutte Contre le Cancer (FNCLCC) grading system used in STS is of prognostic value since low grade MPNST (FNCLCC grade 1) has improved survival (2). However, only 10% of MPNSTs are grade 1, and the FNCLCC grading can likely only distinguish prognosis between grades 1 and 3 (2, 5). Moreover, the histological distinction between low-grade MPNST and benign neurofibroma with atypia is difficult as objective criteria are lacking, causing interobserver variability. In the context of NF1, the diagnosis of progression to MPNST is even more challenging. Recently, a consensus view has been published defining “atypical neurofibromatous neoplasm of uncertain biologic potential (ANNUBP)” as an intermediate lesion in NF1 patients (14). While driver mutations are increasingly being studied, the transition of neurofibromas to MPNSTs is not yet fully understood. Clinical parameters as predictors of outcome have been studied more commonly, but independent predictors are found inconsistently (3). Although radiation-induced MPNSTs have repeatedly been associated with worse survival, the influence of NF1 disease on survival has been subject of debate (3, 13, 15). Better classification systems for MPNSTs are therefore urgently needed.

Currently, surgery remains the only proven treatment to improve survival (1–3). Chemotherapy has limited effect in localized disease, and its use is controversial. Some studies suggest a minor benefit in high-grade, large, and deep MPNST (16–18). Moreover, 10–20% of patients present with metastatic or unresectable disease and up to 50% of patients will develop metastases over time (1–5, 13, 19). Targeted therapies are warranted, but so far none have been proven effective (20). Immunohistochemical and genetic markers may predict more aggressive behavior in MPNSTs, but their association with oncological outcome has uncommonly been investigated and few are yet used in daily practice for prognostication. For this reason this systematic review set out to summarize current knowledge on the prognostic value of immunohistochemical and genetic markers. Such markers may enhance prognostication and aid in elucidating driver mutations of malignancy.

## Methods

### Literature Search

A systematic search was performed in Embase and PubMed databases according to the Preferred Reporting Items for Systematic Reviews and Meta-analysis (PRISMA) guidelines, in order to identify all potentially relevant articles as of March 2020. The string was built with the help of a professional librarian using search terms related to ‘MPNST’ and ‘prognostic’. The exact search syntaxes for PubMed and Embase are shown in **Supplementary Table 1**. Studies were included that evaluated the association of immunohistochemical markers and genetic alterations to oncological outcomes in MPNST patients. Exclusion criteria included lack of full text or studies without specific analyses fitting our inclusion criteria. The initial review was conducted by two independent authors (EM. and IA). Disagreements were solved through discussion in which one additional author was involved (CV).

### Data Extraction and Synthesis

Data extracted from studies included: study period, total number of patients, mean age and range, percentage NF1 patients, markers and genetic alterations investigated for prognostic value, and analyses used to identify prognosticators. For all markers and genetic alterations investigated additional information was extracted: number of patients with survival data, population with ‘positive’ test, oncological outcome analyzed, and whether its prognostic value was corrected for common clinical prognostic factors. Whenever the marker was independently associated with outcome, the hazard ratio was noted. Common factors for which could have been adjusted in multivariable models included: age, presence of NF1, tumor size, tumor site, metastasis at diagnosis, tumor depth, tumor grade, and surgical margin (3). All results of the predictive value of markers were presented or re-calculated to represent the marker cut-off as a negative predictor of survival. Qualitative synthesis was performed for all studies, summarizing results based on type of analysis. Immunohistochemical markers were further stratified into markers of differentiation, receptors and their ligands, Ras pathway, cell cycle regulation, p53 pathway, vascularization, and others. For each immunohistochemical marker cumulative incidence of univariable and multivariable association to survival (disease-specific or overall) or disease-free survival (recurrence, metastasis, or both) were calculated.

## Results

**Figure 1:**
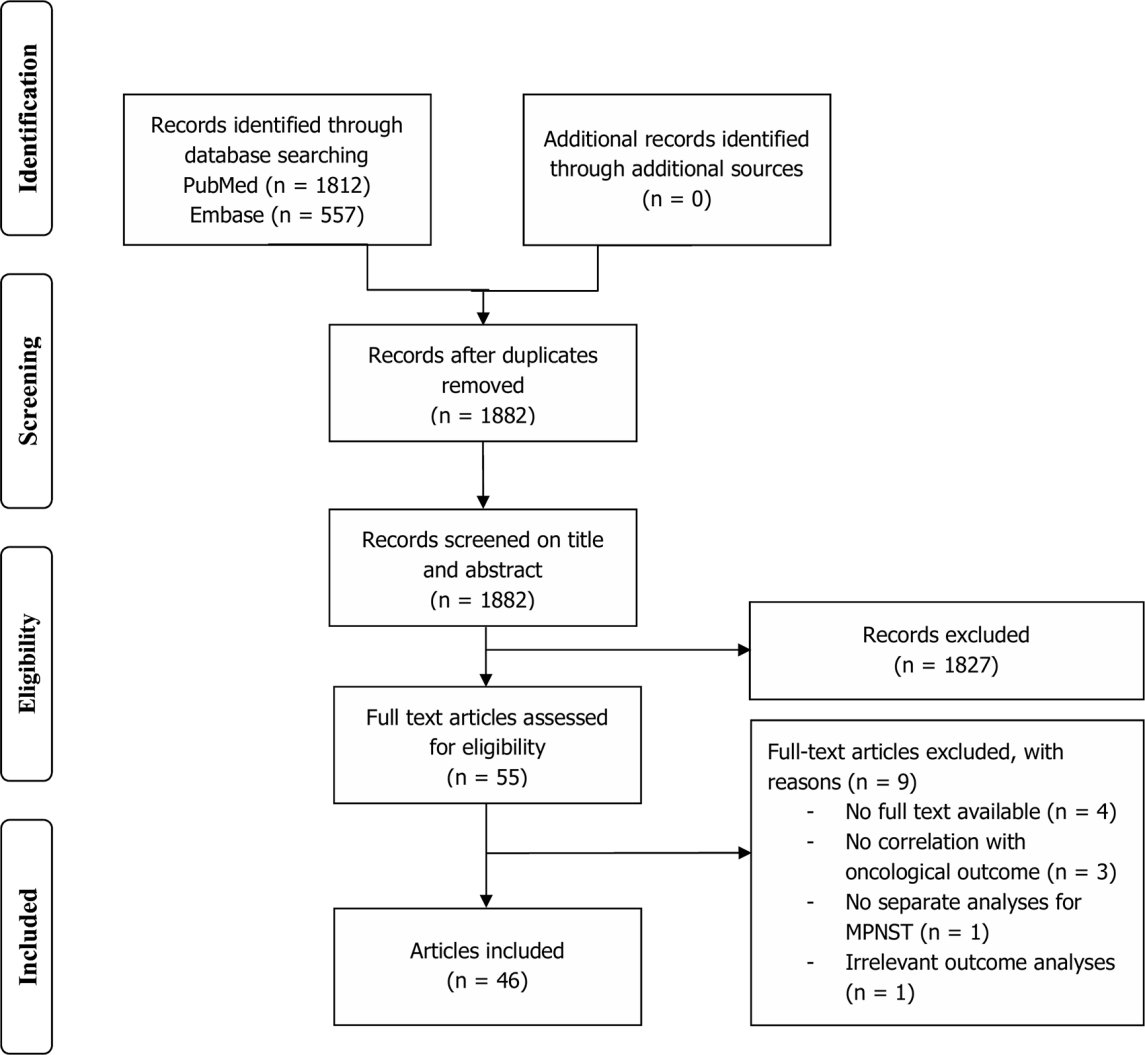
Flowchart depicting study selection.

**Figure 2:**
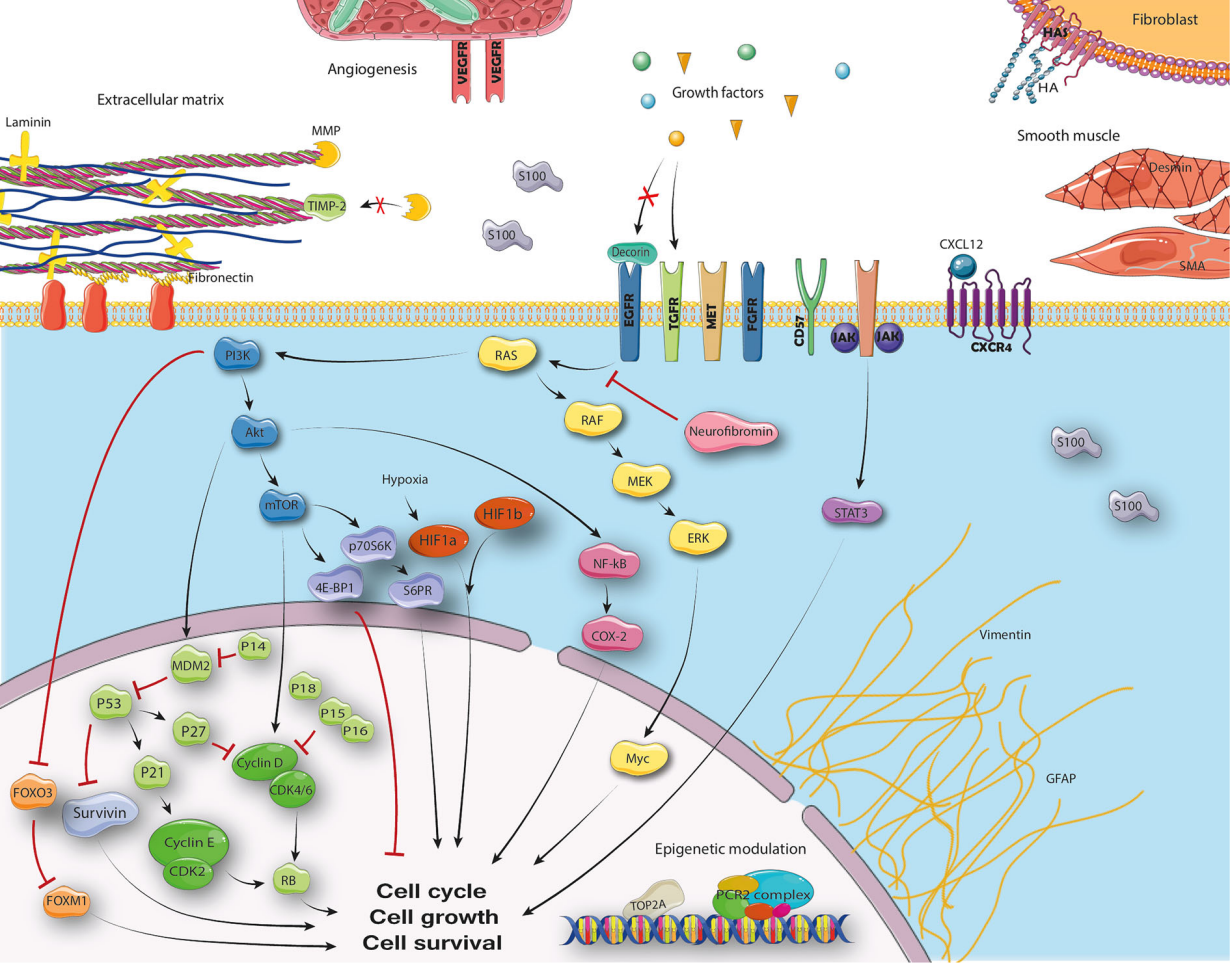
Cellular pathways in MPNST.

After removal of duplicates, a total of 1,882 articles were identified in PubMed and Embase databases (**Figure 1**). Title and abstract screening resulted in 55 potentially relevant articles, of which 46 were selected for qualitative synthesis after full-text screening. Mean age differed between 11 and 50 years old (range of all patients 1–94). Prevalence of NF1 patients in study populations ranged from 0 to 100% (mean: 48.0%). Immunohistochemical markers were studied exclusively in 36 studies, genetic alterations in seven studies, and both in three studies (**Table 1**). A total of 67 different immunohistochemical markers and numerous genetic alterations were evaluated (**Table 2**, **Figure 2**).

**Table 1:** Study characteristics of included studies.

Author, year	Study period	N	Age (range)	NF1	Markers and genetic alterations	Analysis type^a^
Alaggio et al. (21)	1990–2007	35	11 (1–18)	42.9%	*BIRC5*	RT-PCR
Benassi et al. (22)	*NA*	17	*NA*	*NA*	Laminin receptor	IHC
Benassi et al. (23)	*NA*	15	*NA*	*NA*	MMP-2, MMP-9, TIMP-2	IHC
Brekke et al. (24)	1980–2002	64	41 (13–85)	43.8%	p53, p-RB, CDK2, CDK4, cyclin D1, cyclin D3, cyclin E1, p14, p16, p18, p21, p27, MDM2, Ki67	IHC
Brekke et al. (25)	1980–2002	48	37 (11–79)	58.3%	Xq loss, 10q loss, 16p gain, 16q loss, 5p gain, 2q gain, 6q gain, 7q gain, Xp loss, 10p loss, 4q loss, 20q gain, 1q gain	aCGH
Cleven et al. (26)	1979–2007	162	*NA*	49.4%	H3K27me3	IHC
Danielsen et al. (27)	1973–2008	91	48 (11–79)	48.4%	*RASSF1A*	Methylation- specific PCR
Endo et al. (28)	1964–2008	99	*NA*	33.3%	p14, p15, p16, Ki67	IHC
Endo et al. (7)	1964–2010	88	*NA*	40.9%	p-Akt, p-mTOR, p-S6RP, p-p70S6K, p-4E-BP1, p-MEK, p-ERK, Ki67	IHC
Fan et al. (29)	*NA*	56	*NA*	*NA*	MET, MDM2, p53	IHC
Fukushima et al. (30)	1964–2011	82	*NA*	39.0%	HIF-1α, HIF-2α, MVD, Ki67	IHC
Gong et al. (31)	2006–2015	14	46 (23–66)	21.4%	Ki67	IHC
Hakozaki et al. (32)	1992–2008	44	50 (15–86)	47.7%	COX-2	IHC
Halling et al. (33)	*NA*	28	39 (15–84)	50.0%	p53	IHC
Høland et al. (34)	1980–2010	100	36 (11–82)	50.0%	*TP53*, *MDM2*	aCGH, RT-PCR
Holtkamp et al. (35)	*NA*	36	40 (13–78)	61.1%	MMP-13, p53 codon	PCR, IHC
Holtkamp et al. (36)	*NA*	34	*NA*	76.5%	*CDKN2A*	MLPA
Ikuta et al. (37)	1986–2011	30	45 (17–77)	53.3%	HA, HAS1, HAS2, HAS3	IHC
Jia et al. (38)	2002–2011	30	49 (11–71)	*NA*	Decorin	IHC
Keizman et al. (39)	1994–2006	51	41 (*NA*)	51.0%	EGFR	IHC
Kobayashi et al. (40)	1964–2004	96	43 (0–86)	41.2%	CHFR, Ki67	IHC
Kolberg et al. (41)	1980–2002	63	33 (13–85)	44.4%	Survivin, TK1, TOP2A	IHC
Kourea et al. (42)	*NA*	35	*NA* (*NA*)	*NA*	p53, p-RB, p21, p27, cyclin D1, cyclin E, Ki67	IHC
Krawczyk et al. (43)	1992–2013	26	10 (*NA*)	34.6%	Survivin, cyclin D1, osteopontin, fibronectin, p53	IHC
Kresse et al. (44)	*NA*	7	47 (24–78)	*NA*	17q23.2-q25.3, *TOP2A*, *ETV4*, *HOXB7*, *BIRC5*, miR142p-3p, miR142-5p, miR201, miR21, miR338	aCGH, RT-PCR
LaFemina et al. (45)	1982–2011	105	38 (16–87)	40.0%	S100	IHC
Le Guellec et al. (46)	1990–2013	124	37 (7–94)	54.8%	S100, MDM2, desmin	IHC
Leroy et al. (47)	1988–1999	17	32 (17–56)	100%	p53	IHC
Lu et al. (48)	1990–2012	74	39 (11–79)	58.1%	ATRX	IHC
Meis et al. (49)	1965–1985	70	10 (0–15)	20.5%	S100	IHC
Nobeyama and Nakagawa (50)	*NA*	20	(15–70)	100%	*MAGEA3*	Methylation-specific PCR
Otsuka et al. (51)	1975–2016	145	48 (1–88)	29.7%	H3K27me3	IHC
Panse et al. (52)	*NA*	39	*NA*	*NA*	p-STAT3	IHC
Pekmezci et al. (53)	1991–2012	39	37 (11–72)	66.7%	H3K27me3	IHC
Skotheim et al. (54)	1980–2000	51	50 (20–86)	37.3%	TOP2A, Ki67	IHC
Tabone-Eglinger et al. (55)	1985–2005	52	23 (3–60)	50.0%	EGFR	IHC
Torres et al. (56)	1986–2006	96	*NA*	57.3%	MET, HGF, p-MET, p53, S100	IHC
de Vasconcelos et al. (57)	1990–2010	29	*NA* (22–83)	58.6%	MCD, MVD, Ki67	IHC
Wang et al. (58)	2001–2012	43	49 (*NA*)	14.0%	S100, vimentin, GFAP, NSE, Ki67, SMA, CD57	IHC
Wasa et al. (59)	1987–2006	22	43 (16–83)	50.0%	VEGF, MVD	IHC
Watanabe et al. (60)	*NA*	49	41 (17–86)	44.9%	p53, Ki67, MDM2, p21	IHC
Yu et al. (61)	*NA*	123	*NA*	38.2%	*SOX5, NOL1, MLF2, FOXM1, FKBP4, CDK4, TSPAN31, ERBB2, MYC, TP53*, SOX5, FOXM1, Myc, p53	aCGH, RT-PCR, FISH, IHC
Yuan et al. (62)	1999–2016	159	40 (5–76)	44.0%	S100, Ki67, vimentin, NF, GFAP	IHC
Zhang et al. (63)	1991–2011	58	47 (6–86)	0.0%	CXCR4, CXCL12, cyclin D1	IHC
Zhou et al. (64)	*NA*	63	*NA*	*NA*	FGFR1, FGFR2, FGFR4, *FGFR1*	FISH, IHC
Zou et al. (4)	1986–2006	140	35 (1–80)	51.4%	S100, Ki67, p53, VEGF, EGFR, p-MEK	IHC

^a^Used for correlation with outcome.

4E-BP1, eukaryotic translation initiation factor 4E-binding protein 1; aCGH, array-based comparative genomic hybridization; CDK, cyclin dependant kinase; CHFR, checkpoint with forkhead-associated domain and ring finger; COX-2, cyclooxygenase-2; CXCR4, C-X-C motif chemokine receptor 4; CXCL12, C-X-C motif chemokine ligand 12; EGFR, epidermal growth factor receptor; ERK, extracellular signal-regulated kinases; FISH, fluorescence in situ hybridization; FGFR, fibroblast growth factor receptor; FOXM1, forkhead box protein M1; GFAP, glial fibrillary acidic protein; H3K27me3, trimethylation of lysine 27 of histone H3; HA, hyaluronan; HAS, hyaluronan synthase; HIF, hypoxia-inducible factor; IHC, immunohistochemistry; MCD, mast cell density; MDM2, mouse double minute 2 homolog; MEK, mitogen-activated protein kinase kinase; Met, metastasis; MLPA, multiplex ligation-dependent probe amplification; MMP-13, matrix metallopeptidase 13; mTOR, mammalian target of rapamycin, MVD, microvessel density; N, total number of patients; NA, not available; NF1, neurofibromatosis type 1p-, phosphorylated; RASSF1A, Ras association domain family member 1, isoform A; RT-PCR, reverse transcription polymerase chain reaction; S6RP, ribosomal protein S6; SMA, smooth muscle actin; STAT3, Signal transducer and activator of transcription 3; TIMP-2, tissue inhibitor of metalloproteinase 2; TK1, thymidine kinase 1; TOP2A, topoisomerase 2-alpha; VEGF, vascular endothelial growth factor.

**Table 2:** Prognostic value of immunohistochemical markers.

Marker	N	Survival^a^				N	Disease-free survival^a^			
		Univariate	Multivariate				Univariate	Multivariate		
		+	*NsA*	+	−		+	*NA*	+	−
*Differentiation*										
S100	7	57%	25%	50%	25%	4	50%	50%	50%	0%
GFAP	2	0%	*NA*	*NA*	*NA*	2	0%	*NA*	*NA*	*NA*
Vimentin	2	0%	*NA*	*NA*	*NA*	2	0%	*NA*	*NA*	*NA*
NSE	1	0%	*NA*	*NA*	*NA*	1	0%	*NA*	*NA*	*NA*
SMA	1	0%	*NA*	*NA*	*NA*	1	100%	0%	0%	100%
Desmin	1	0%	*NA*	*NA*	*NA*	1	0%	*NA*	*NA*	*NA*
CD57	1	0%	*NA*	*NA*	*NA*	1	100%	0%	0%	100%
*Vascularization*										
MVD	4	25%	0%	100%	0%	0	*NA*	*NA*	*NA*	*NA*
VEGF	2	50%	100%	0%	0%	0	*NA*	*NA*	*NA*	*NA*
*Receptors and ligands*										
EGFR	3	67%	100%	0%	0%	1	100%	100%	0%	0%
MET	2	0%	*NA*	*NA*	*NA*	1	0%	*NA*	*NA*	*NA*
p-MET	1	100%	0%	100%	0%	0	*NA*	*NA*	*NA*	*NA*
HGF	1	0%	*NA*	*NA*	*NA*	0	*NA*	*NA*	*NA*	*NA*
CXCR4	1	100%	0%	0%	100%	1	0%	*NA*	*NA*	*NA*
CXCL12	1	0%	*NA*	*NA*	*NA*	1	0%	*NA*	*NA*	*NA*
FGFR1	1	100%	0%	100%	0%	1	0%	*NA*	*NA*	*NA*
FGFR2	1	0%	*NA*	*NA*	*NA*	1	0%	*NA*	*NA*	*NA*
FGFR4	1	0%	*NA*	*NA*	*NA*	1	100%	0%	0%	100%
HA	1	100%	0%	0%	100%	1	100%	0%	100%	0%
HAS1	1	0%	*NA*	*NA*	*NA*	1	0%	*NA*	*NA*	*NA*
HAS2	1	0%	*NA*	*NA*	*NA*	1	0%	*NA*	*NA*	*NA*
HAS3	1	0%	*NA*	*NA*	*NA*	1	0%	*NA*	*NA*	*NA*
Decorin	1	100%	100%	0%	0%	0	*NA*	*NA*	*NA*	*NA*
*Ras pathway*										
p-MEK	2	0%	*NA*	*NA*	*NA*	0	*NA*	*NA*	*NA*	*NA*
NF	1	0%	*NA*	*NA*	*NA*	1	0%	*NA*	*NA*	*NA*
p-ERK	1	0%	*NA*	*NA*	*NA*	0	*NA*	*NA*	*NA*	*NA*
p-Akt	1	0%	*NA*	*NA*	*NA*	0	*NA*	*NA*	*NA*	*NA*
p-mTOR	1	100%	0%	100%	0%	0	*NA*	*NA*	*NA*	*NA*
p-p70S6K	1	0%	*NA*	*NA*	*NA*	0	*NA*	*NA*	*NA*	*NA*
p-4E-BP1	1	0%	*NA*	*NA*	*NA*	0	*NA*	*NA*	*NA*	*NA*
p-S6RP	1	100%	0%	100%	0%	0	*NA*	*NA*	*NA*	*NA*
COX-2	1	100%	0%	100%	0%	0	*NA*	*NA*	*NA*	*NA*
Myc	1	100%	0%	0%	100%	0	*NA*	*NA*	*NA*	*NA*
*Cell cycle regulation*										
p53	10	40%	25%	75%	0%	3	67%	50%	50%	0%
MDM2	4	0%	*NA*	*NA*	*NA*	2	50%	100%	0%	0%
Cyclin D1	4	25%	0%	100%	0%	3	33%	0%	100%	0%
p21	3	0%	*NA*	*NA*	*NA*	1	0%	*NA*	*NA*	*NA*
Cyclin E	2	0%	*NA*	*NA*	*NA*	1	0%	*NA*	*NA*	*NA*
p-RB	2	0%	*NA*	*NA*	*NA*	1	100%	100%	0%	0%
p14	2	100%	0%	50%	50%	0	*NA*	*NA*	*NA*	*NA*
p16	2	50%	0%	100%	0%	0	*NA*	*NA*	*NA*	*NA*
p27	2	50%	100%	0%	0%	1	0%	*NA*	*NA*	*NA*
p15	1	0%	*NA*	*NA*	*NA*	0	*NA*	*NA*	*NA*	*NA*
p18	1	0%	*NA*	*NA*	*NA*	0	*NA*	*NA*	*NA*	*NA*
FOXM1	1	100%	0%	100%	0%	0	*NA*	*NA*	*NA*	*NA*
SOX5	1	0%	*NA*	*NA*	*NA*	0	*NA*	*NA*	*NA*	*NA*
CDK2	1	0%	*NA*	*NA*	*NA*	0	*NA*	*NA*	*NA*	*NA*
CDK4	1	0%	*NA*	*NA*	*NA*	0	*NA*	*NA*	*NA*	*NA*
Cyclin D3	1	0%	*NA*	*NA*	*NA*	0	*NA*	*NA*	*NA*	*NA*
HIF1α	1	100%	0%	100%	0%	0	*NA*	*NA*	*NA*	*NA*
HIF2α	1	0%	*NA*	*NA*	*NA*	0	*NA*	*NA*	*NA*	*NA*
CHFR	1	100%	0%	100%	0%	0	*NA*	*NA*	*NA*	*NA*
*Epigenetic modulation*										
H3K27me3	3	67%	0%	50%	50%	1	0%	*NA*	*NA*	*NA*
TOP2A	2	100%	50%	0%	50%	1	100%	100%	0%	0%
*Other*										
Ki67	13	62%	0%	25%	75%	5	40%	0%	50%	50%
Survivin	2	50%	0%	0%	100%	1	0%	*NA*	*NA*	*NA*
ATRX	1	100%	0%	100%	0%	0	*NA*	*NA*	*NA*	*NA*
TK1	1	100%	0%	0%	100%	0	*NA*	*NA*	*NA*	*NA*
MCD	1	0%	*NA*	*NA*	*NA*	0	*NA*	*NA*	*NA*	*NA*
p-STAT3	1	100%	100%	0%	0%	1	0%	*NA*	*NA*	*NA*
Osteopontin	1	0%	*NA*	*NA*	*NA*	1	0%	*NA*	*NA*	*NA*
Fibronectin	1	0%	*NA*	*NA*	*NA*	1	0%	*NA*	*NA*	*NA*
MMP-2	0	*NA*	*NA*	*NA*	*NA*	1	0%	*NA*	*NA*	*NA*
MMP-9	0	*NA*	*NA*	*NA*	*NA*	1	0%	*NA*	*NA*	*NA*
MMP-13	0	*NA*	*NA*	*NA*	*NA*	1	0%	*NA*	*NA*	*NA*
TIMP-2	0	*NA*	*NA*	*NA*	*NA*	1	0%	*NA*	*NA*	*NA*
Laminin receptor	0	*NA*	*NA*	*NA*	*NA*	1	100%	100%	*NA*	*NA*

^a^Univariate analysis: significant effect (+), not significant effect (**−**); Multivariate analysis: not performed (NA), significant effect (+), nog significant effect (**−**).

4E-BP1, eukaryotic translation initiation factor 4E-binding protein 1; CDK, cyclin dependent kinase; CHFR, checkpoint with forkhead-associated domain and ring finger; COX-2, cyclooxygenase-2; CXCR4, C-X-C motif chemokine receptor 4; CXCL12, C-X-C motif chemokine ligand 12; DFS, disease-free survival (either time to recurrence, metastasis, or both); EGFR, epidermal growth factor receptor; ERK, extracellular signal-regulated kianses; FGFR, fibroblast growth factor receptor; FOXM1, forkhead box protein M1; GFAP, glial fibrillary acidic protein, H3K27me3, trimethylation of lysine 27 of histone H3; HA, hyaluronan; HAS, hyaluronan synthase; HIF, hypoxia-inducible factor; MCD, mast cell density; MDM2, mouse double minute 2 homolog, MEK, mitogen-activated protein kinase kinase; MMP, matrix metalloproteinase; mTOR, mammalian target of rapamycin; MVD, microvessel density; N, number of studies, NA, not applicable; NF, neurofibromin; p-, phosphorylated, S, survival (either disease-specific or overall); S6RP, ribosomal protein S6; SMA, smooth muscle actin; STAT3, Signal transducer and activator of transcription 3; TIMP-2, tissue inhibitor of metalloproteinase 2; TK1, thymidine kinase 1, TOP2A, topoisomerase 2-alpha; VEGF, vascular endothelial growth factor.

### Differentiation

Seven mesenchymal and neuronal differentiation markers were evaluated (**Table 2**), most commonly S100 (4, 45, 46, 56, 58, 62). In univariable analysis complete absence of S100 was found negatively associated with survival in four/six studies. Two studies showed the absence of S100 to be an independent predictor of worse survival with HR 4.5 (95% CI: 2.0–12.1) and HR 6.6 (95% CI: 1.8–23.8) (4, 62). All seven markers were also evaluated for association with disease-free survival (DFS). Negative S100 staining was associated with worse DFS in two/four studies, of which one study showed an independent association (HR 4.2, 95% CI: 1.5–12.3) (62). Negative smooth muscle actin (SMA) and CD57 staining were also found associated with worse DFS in univariable analysis in one study, but not in multivariable analysis (58).

### Vascularization

Microvascular density (MVD) and vascular epithelial growth factor (VEGF) staining were evaluated as vascularization markers (**Table 2**) (4, 28, 30, 57, 59). High MVD was associated with worse survival in one/four studies. This association was also significant in multivariable analyses (HR 7.3, 95% CI: 1.4–38.5) (57). High VEGF staining was associated with worse survival in one/two studies, but this was not studied in a multivariable model (59). No markers were studied for association with DFS.

### Receptors and Ligands

Immunohistochemical expression of nine different receptors or their ligands were evaluated, most commonly the epidermal growth factor receptor (EGFR, **Table 2**) (4, 29, 37–39, 55, 56, 63, 64). Increased EGFR staining was associated with worse survival in univariable analysis in two/three studies, but this was not evaluated in a multivariable model (4, 39, 54). Increased phosphorylated MET (p-MET), C-X-C motif chemokine receptor 4 (CXCR4), and low fibroblast growth factor receptor 1 (FGFR1) staining were also associated with worse survival in univariable analysis, but only p-MET (HR 1.04, 95% CI: 1.0–1.1) and FGFR1 (HR 2.8, 95% CI: 1.2–6.7) were independently associated with survival (37, 56, 63, 64). Increased EGFR and FGFR4 were associated with worse DFS, but only in univariable analyses (39, 64). On a genetic level, no amplification of *FGFR1* on fluorescence *in situ* hybridization (FISH) was associated with worse survival and DFS in univariable analysis (**Supplementary Table 2**) (64). Copy number alterations in *ERBB2* were not associated with survival (61).

### Extracellular Matrix

Twelve extracellular matrix markers were studied, of which none was evaluated more than once (**Table 2**) (22, 23, 35, 37, 38, 43). Only increased hyaluronan (HA) and decorin staining were associated with decreased survival, but none in a multivariable model (36, 37). Increased HA and laminin receptor were associated with worse DFS, but only HA was associated with worse DFS in a multivariable model (HR 5.7, 95% CI: 1.2–26.4) (22, 37).

### Ras Pathway

Ten different Ras pathway proteins were stained, but only phosphorylated MAPK kinase (MEK) was evaluated more than once (**Table 2**) (4, 7, 32, 61, 62). Increased phosphorylated mammalian target of rapamycin (p-mTOR), phosphorylated ribosomal protein S6 (p-S6RP), cyclooxygenase-2 (COX-2), and Myc staining were associated with worse survival univariable analysis (7, 32, 61). Only increased p-mTOR (HR 2.6, 95% CI: 1.3–5.5), p-S6RP (HR 2.5, 95% CI: 1.3–5.5), and COX-2 (HR 3.0, 95% CI: 1.1–10.2) staining were independently associated with worse survival (7, 32). No Ras pathway associated immunohistochemical marker was found associated with DFS. On a genetic level, copy number alterations of *MYC* were not associated with survival (61). Methylation of *RASSF1A* gene was associated independently with worse survival in one study (HR 5.2, 95% CI: 1.4–19.4, **Supplementary Table 2**) (27). This association was however only found in the NF1 subpopulation.

### Cell Cycle Regulation

Sixteen immunohistochemical markers of cell cycle regulation were evaluated, most commonly p53 (**Table 2**) (4, 24, 26, 28, 29, 33, 40, 42, 43, 46, 47, 51, 53, 56, 60, 61, 63). Low p14, p16, checkpoint with forkhead-associated domain and ring finger (CHFR), and increase in p53, p14, cyclin D1, p27, and forkhead box protein M1 (FOXM1) staining were associated with worse survival in univariable analysis (4, 26, 28, 40, 42, 43, 51, 56, 61). Positive p53 staining was independently associated with survival in three/four studies (HR 1.8, 95% CI: 1.0–3.3, HR 2.3, 95% CI: 1.2–4.5, and HR 6.4, 95% CI: 1.5–29.0) (4, 24, 56). Increased staining of cyclin D1 (HR 15.9, 95% CI: 2.0–125.0), HIF1*α* (HR 8.3, 95% CI: 2.8–28.9), FOXM1 (HR 1.9, 95% CI: 1.1–3.3), and decreased staining of p16 (HR 2.2, 95% CI: 1.5–3.2) and p14 (HR 2.7, 95% CI: 1.8–4.2) were also independently associated with worse survival in one study each (28, 30, 43, 61). Positive staining of p53, MDM2, cyclin D1, and p-RB were associated with worse DFS in univariable analysis (29, 42, 43). Only cyclin D1 (HR 11.1, 95% CI: 2.8–47.6) and p53 (HR 3.2, 95% CI: 1.0–10.4) were independently associated with worse DFS in one study (43). On a genetic level, mutation, homozygous loss, or loss of heterogeneity of *TP53* was associated with worse survival in two/three studies (**Supplementary Table 2**) (34, 35, 61). The copy number gain of *MDM2* and *CDK4* as well as amplification on FISH of *CDK4* was associated with worse survival (34, 61). Gain (HR 4.2, 95% CI: 1.4–12.4) or amplification (HR 2.0, 95% CI: 1.0–4.0) of *CDK4* was independently associated with worse survival (59). The combination of either *MDM2* gain or *TP53* aberration made a high risk group (16%) for worse survival with a HR 3.4 (95% CI: 1.4–8.3) (34). In the same study, a gene expression profile was made and a score of ≥0.12 was present in 66.7% of the population which was associated with worse survival as well (HR 4.0, 95% CI: 1.3–12.1). Another study on DNA copy number changes found a significant association with worse survival for gain at 17q23.2–25.3, but not in several related genes or micro-RNAs in this region (44). The association was not evaluated in a multivariable model. A gain in *FOXM1* was worse survival in another study (61). Only the polymorphism of p53Pro^72^ was associated with worse DFS in one study (35). This association was not evaluated in a multivariable model.

### Epigenetic Modulation

Two epigenetic modulating proteins were investigated as immunohistochemical markers (**Table 2**) (26, 41, 51, 53, 54). Loss of trimethylation of lysine 27 of histone H3 (H3K27me3) and increased topoisomerase 2-alpha (TOP2A) staining were both associated with decreased survival (26, 41, 51, 54). Only H3K27me3 was independently associated with worse survival (HR 2.6, 95% CI: 1.2–5.7) in one out of two studies (26, 51). Increased TOP2A staining was also associated with worse DFS in one study (54). High copy number changes of *TOP2A* was not associated with worse survival (**Supplementary Table 2**) (44).

### Other

Thirteen other immunohistochemical markers were studied, most commonly the proliferation marker Ki67 (4, 7, 24, 30, 31, 40–43, 48, 52, 54, 57, 58, 60–62). On average a cut-off at 20.9% (range: 5–30%) for high Ki67 staining was used and it was significantly associated with worse survival in 8/12 studies, of which two studies showed an independent association (HR 2.4, 95% CI: 1.1–4.9 and HR 10.2, 95% CI: 3.6–32.1) (28, 30). Increased survivin, thymidine kinase 1 (TK1), phosphorylated signal transducer and activator of transcription 3 (p-STAT3), and hypoxia-induced factor 1-alpha (HIF1*α*) and decreased ATRX staining were associated with worse survival (30, 41, 48). Both decreased ATRX (HR 5.3, 95% CI: 1.4–20.4) and positive HIF1*α* staining (HR 8.3, 95% CI: 2.8–28.9) were independently associated with worse survival (30, 48). One study showed that when there was high survivin and high TK1 staining or low survivin and high TOP2A staining a high risk group of patients could be stratified with HR 4.6 (95% CI: 1.5–14.4) (41). Increased staining of Ki67 and laminin receptor were associated with worse DFS (22, 31, 62). Only high Ki67 staining was shown to have an independent association with worse DFS in one/two studies (HR 3.8, 95% CI: 1.7–8.5) (62). Four studies investigated several other genetic alterations, including two on *BIRC5*, the gene encoding surviving (21, 25, 44, 50). One out of two studies showed that an increase in *BIRC5* mRNA was associated with worse survival in univariable analysis (21). Gain at 17q23.2–25.3 was associated with worse survival in univariable analysis in another study (44). One study investigated the effect of chromosomal gains and losses and showed an independent effect on worse survival for Xq loss (HR 3.6, 95% CI: 1.6–8.3), 10q loss (HR 3.2, 95% CI: 1.4–7.7), and 16p gain (HR 2.5, 95% CI: 1.0–6.2) (25). Together a high risk group (63% of population) was obtained for either gain or loss which resulted in a HR 11.0 (95% CI: 3.5–35.0) after correction for several clinical characteristics. A gain in *SOX5* and *NOL1* were associated with worse survival in one study, but only in univariable analyses (61). Finally, methylation of *MAGEA3* was also associated with worse survival in univariable analysis (50).

## Discussion

he underlying biology of MPNSTs remains complex as is highlighted by the diverse findings of studies included in this review. Many markers and genetic alterations have been proposed to be of prognostic value, yet outcomes are infrequently repeated. Alterations in *TP53* or its resulting increased p53 staining were commonly found associated with survival and DFS as were several other proteins and genes involved in cell cycle regulation. Epigenetic modulatory proteins, especially loss of H3K27me3, and more general markers as absence of S100 and increased Ki67 were commonly found to be of prognostic value too.

### Prognostication in MPNST

The predictive value of clinical parameters including patient and tumor characteristics has been studied more commonly than immunohistochemical or genetic biomarkers in MPNST. Increasing age, large tumor size, metastatic disease at diagnosis, and tumors not amenable to complete resection are the most commonly found predictors of worse survival in MPNST (2, 3, 5, 13, 45, 65). This emphasizes the importance of early diagnosis of MPNST in order to completely resect tumors, along with finding new systemic therapies to improve the prognosis of irresectable and metastatic disease. Non-extremity tumor sites have also been shown to have a negative impact on survival; however, this may be more true for those arising in retroperitoneal or pelvic sites (1, 3, 5, 13, 66). Tumor depth used to be incorporated for prognostication in the AJCC staging system for STS, but has varyingly been shown to be of prognostic value in MPNST (2, 3, 5, 13, 45, 65). The importance of NF1 disease has also been subject of debate. A meta-analysis in 2012 showed no difference in survival for patients in papers published after 2000 (15). However, recent large cohorts did find an independent association with worse survival for NF1 patients (3, 13, 67, 68). Altogether, clinical parameters seem to be able to predict some part of a patient’s course of disease. The addition of tumor biology to clinical parameters may further increase our ability to stratify subgroups of patients based on prognosis. *TP53* is one of the few recurrently mutated genes found in MPNST. *TP53* mutations and high p53 staining were independently associated with survival or DFS in five different studies (4, 24, 34, 43, 56). This may indicate that aberrations in this gene may indeed be of clinical importance. Other genes involved in cell cycle regulation such as *CDKN2A* and downstream proteins are commonly altered and may not only contribute to tumorigenesis but also be of clinical significance, supporting a belief that dysregulations in this cellular pathway are of overall importance. Loss of polycomb regressive complex 2 (PRC2) complex has recently been shown to be common in MPNSTs due to mutations in *EED* and *SUZ12* (9, 69). This results in loss of H3K27me3 which can reliably distinguish high-grade MPNSTs from their benign counterparts by immunohistochemistry (26, 70). MPNSTs without loss of H3K27me3 staining may also be associated with less aggressive behavior as many low-grade MPNSTs are known to retain this expression (14, 26). Preclinical research on targeted therapies has most frequently shown promising results targeting proteins in the Ras pathway, especially when combined with other target drugs, but unfortunately no clinical trial has proven benefit to date (20). Activated proteins in the Ras pathway, including p-mTOR, p-4E-BP1, p-S6RP, COX-2, and Myc as well as methylation of *RASSF1A* may however predict worse survival (6, 7, 27, 32). Targeting vascular pathways in MPNSTs may be beneficial, but unfortunately few studies have focused on this. Studies included in this review also showed that increased vascularity, as evidenced by increased microvascular density as well as increased expression of VEGF, may be associated with more aggressive biological behavior (57, 59). It seems that many other targets may be of prognostic value as well emphasizing the need for further research into MPNST tumor biology. Survivin markers may for instance stratify a subgroup of patients and survivin has been shown a viable target in a xenograft mouse model (71). Seeing as MPNSTs are heterogenic and markers such as p53 are not be MPNST specific, combined scores of different markers and genetic alterations may be of most clinical importance. Four studies in this review highlight this phenomenon demonstrating increased prognostic value when markers are combined (25, 28, 34, 41).

### Strengths and Limitations

Unfortunately, due to the large heterogeneity of published studies meta-analyses were not presumed feasible. All studies included in this review were retrospective of nature inherently harboring bias. None of the markers and genetic alterations found in these studies were prospectively validated. Moreover, many did not evaluate the prognostic value of their markers in a multivariable model nor on their discriminative ability. Studies that evaluated the prognostic value of markers in a multivariable model were nonetheless not always capable to correct for all common clinical variables. MPNSTs are rare sarcomas, which in combination with their complex biology, make it difficult to obtain enough cases to create valuable models. But as shown in this review, several markers and genetic alterations may already be of clinical importance as they have shown an independent association with survival in addition to clinical parameters. Future research should therefore be encouraged to replicate these results using larger datasets obtained by large-scale international collaborations. Important immunohistochemical staining may include Ki67, S100, p53, and H3K27me3 in all patients, and possibly further staining of proteins associated with cell cycle regulation. In turn individual prediction models for MPNST patients specifically may arise taking their significant heterogeneity into account. Such models may better elucidate patient selection for (neo)adjuvant treatment and targeted therapies, which should then be validated in a prospective database. But as MPNSTs remain rare entities one may also turn to exploratory analyses using machine learning techniques on large STS genetic databases to identify attractive genes as biomarkers or prognostic markers in subtypes of STS (72).

## Conclusion

MPNSTs harbor complex and heterogenic biology and currently lack adequate staging systems. Immunohistochemical markers and genetic alterations are varyingly of prognostic value. Absence of S100 and H3K27me3 and increased Ki67 staining were commonly found to be of independent prognostic value alongside of clinical parameters. Alterations in *TP53* or its consequential increase in p53 staining seems to distinguish a subgroup of MPNSTs with worse outcomes. Immunohistochemical staining and associated genetic alterations of proteins involved in cell cycle regulation and the Ras pathway may also help stratifying patients with worse outcomes. Other markers will likely need further evaluation for validation. A combination of markers may increase the prognostic value.

## Data Availability Statement

All datasets presented in this study are included in the article/**Supplementary Material**.

## Author Contributions

Study conceptualization: EM, CV. Study design: EM, IA, DG, JB, CV. Data acquisition: EM, IA. Drafting manuscript: EM, IA. Study supervision: DG, JB, CV. Manuscript reviewing: DG, JB, CV. Approval of final manuscript: EM, IA, DG, JB, CV. All authors contributed to the article and approved the submitted version.

## Conflict of Interest

The authors declare that the research was conducted in the absence of any commercial or financial relationships that could be construed as a potential conflict of interest.
