# Supplementary material for: Prognostic Significance of Immunohistochemical Markers and Genetic Alterations in Malignant Peripheral Nerve Sheath Tumors: A Systematic Review
Source: Front Oncol. 2020 Dec 22;10:594069. doi: 10.3389/fonc.2020.594069 (PMC7783392; doi:10.3389/fonc.2020.594069)
Supplement: Supplementary file 1 [file Table_1.docx]

1. What institution are you from?

2. What is your standard of care for the following:

a) AR medulloblastoma j) disseminated ependymoma
b) HR medulloblastoma k) localized germinoma
c) infant AR medulloblastoma m) disseminated germinoma
d) infant HR medulloblastoma n) localized NGGCT
e) pLGG (1^st^ line) o) disseminated NGGCT
f) pLGG (2^nd^ line) p) craniopharyngioma
g) pHGG q) localized ATRT
i) localized ependymoma r) disseminated ATRT

3. If you indicated your institution uses SJMB12 or SJMB96 for AR childhood medulloblastoma, please clarify:
a) I do not use SJMB as a standard of practice
b) I use SJMB12 exactly as written
c) I used SJMB96 exactly as written
d) modified SJMB03 therapy with 12 mg/m^2^ of cyclophosphamide
e) other

4. For childhood AR medulloblastoma, please indicate the site of boost:
a) posterior fossa
b) tumor bed

5. For childhood AR medulloblastoma, please indicate dose for boost:
a) 54 Gy b) other

6. For children with anaplastic or large cell histology MB, what dose of CSI would you give:
a) 23.4 Gy b) 36 Gy

7. For childhood HR medulloblastoma, please indicate the site of boost:
a) posterior fossa b) tumor bed

8. For childhood HR medulloblastoma, please indicate the dose of boost:

9. For patients with localized germinoma treated per ACNS1123 for chemotherapy, do you give whole ventricular radiation with 24 Gy?
a) Yes b) No c) Other

10. For patients with germinoma treated per ACNS1123 with whole ventricle irradiation, do you add a boost to the tumour bed?
a) Yes b) No

11. If yes to boost, please state the total cumulative dose (Gy):
a) 30
b) 36
c) 40
d) Other

**Supplementary Figure 1.**  The finalized survey that was sent to neuro-oncologists at all Canadian institutions.
